# Associations of symptom combinations with in-hospital mortality of coronavirus disease-2019 patients using South Korean National data

**DOI:** 10.1371/journal.pone.0273654

**Published:** 2022-08-26

**Authors:** Suyoung Jo, Hee-kyoung Nam, Heewon Kang, Sung-il Cho

**Affiliations:** 1 Department of Public Health Science, Graduate School of Public Health, Seoul National University, Seoul, Korea; 2 Institute of Health and Environment, Graduate School of Public Health, Seoul National University, Seoul, Korea; Anglia Ruskin University, UNITED KINGDOM

## Abstract

**Background:**

There are various risk factors for death in coronavirus disease-2019 (COVID-19) patients. The effects of symptoms on death have been investigated, but symptoms were considered individually, rather than in combination, as predictors. We examined the effects of symptom combinations on in-hospital mortality.

**Methods:**

Data from the Korea Disease Control and Prevention Agency were analyzed. A cohort of 5,153 patients confirmed with COVID-19 in South Korea was followed from hospitalization to death or discharge. An exploratory factor analysis was performed to identify symptom combinations, and the hazard ratios (HRs) of death were estimated using the Cox proportional hazard model.

**Results:**

Three sets of symptom factors were isolated for symptom combination. Factor 1 symptoms were cold-like symptoms, factor 2 were neurological and gastrointestinal symptoms, and factor 3 were more severe symptoms such as dyspnea and altered state of consciousness. Factor 1 (HR 1.14, 95% confidence interval [95% CI] 1.01–1.30) and factor 3 (HR 1.25, 95% CI 1.19–1.31) were associated with a higher risk for death, and factor 2 with a lower risk (HR 0.71, 95% CI 0.71–0.96).

**Conclusions:**

The effect on in-hospital mortality differed according to symptom combination. The results are evidence of the effects of symptoms on COVID-19 mortality and may contribute to lowering the COVID-19 mortality rate. Further study is needed to identify the biological mechanisms underlying the effects of symptom combinations on mortality.

## Introduction

A novel coronavirus, severe acute respiratory syndrome coronavirus 2 (SARS-CoV-2), caused the global pandemic of coronavirus disease 2019 (COVID-19), which was first identified in Wuhan, China, at the end of 2019. As of June 28, 2022, about 542 million confirmed cases of COVID-19 and 6.3 million deaths had been reported globally [1]. The average case-fatality rate (CFR) of COVID-19 was about 2–3% worldwide until early 2021, but this is associated with population size [2]. More recently, the global CFR of COVID-19 has been about 1%. When COVID-19 mortality from March to October 2020 was compared to that of other leading causes from March to October 2018, COVID-19 was the third leading cause of death for persons aged 65 to 84 years. Also, it was the second leading cause of death for persons aged 85 years or above following heart disease in the United States [3].

COVID-19 mortality rates are affected by, among other factors, hospital bed capacity and medical resources. High-income regions have more intensive care units and a greater hospital bed capacity than low-income regions [4]. In South Korea, the lack of hospital beds for COVID-19 patients was a problem not only in the early days of the COVID-19 epidemic but also at the outset of each new phase of the pandemic, which led to deaths of patients waiting for hospitalization [5]. Therefore, assessing the characteristics of patients at high-risk for death would facilitate the objective setting of priorities and appropriate allocation of medical resources. In addition, early treatment of patients with viral pneumonia improves outcomes, so identifying high-risk factors and early treatment of patients with such factors would reduce the COVID-19 mortality rate [6].

Prognostic factors of mortality in COVID-19 patients include older age, male sex, obesity, history of diabetes, cardiovascular, cerebrovascular and kidney diseases, socioeconomic deprivation and, air pollution [2]. Symptoms are the first sign of disease progression, and COVID-19 can be asymptomatic or manifest as mild cold-like symptoms, gastrointestinal symptoms, or severe respiratory symptoms. However, few studies have evaluated the associations between initial symptoms and death, and most have been descriptive. Fever, dry cough, fatigue, and dyspnea are general COVID-19 symptoms [7]. In a few studies, individual symptoms were analyzed as risk factors for mortality, but most were of controversial significance, and only dyspnea was a consistent predictor. However, symptoms need to be considered in combination because they can occur concomitantly, rather than individually. Prior studies have aimed to increase the early diagnosis rate by identifying symptom combinations that increase diagnostic sensitivity and specificity for COVID-19 [8,9]. In one study, five symptom combinations were suggested based on factor analysis. However, no in-depth analysis of clinical symptoms was conducted [10]. Individual patients with COVID-19 can have different symptoms [11] and we hypothesized that there would be several symptom combinations, each with a different risk for mortality.

In this study, we identified symptom combinations on admission by explanatory factor analysis (EFA) and assessed their associations with in-hospital mortality among COVID-19 patients. We analyzed risk factors associated with death, based on the presence and number of symptoms, and symptom combinations. We also examined other risk factors at admission that affected mortality. This study will contribute to the identification of high-risk factors to appropriately allocate medical resources and decrease the mortality rate by identifying initial symptoms predictive of in-hospital mortality in COVID-19 patients.

## Materials and methods

### Study design and data sources

**Fig 1:**
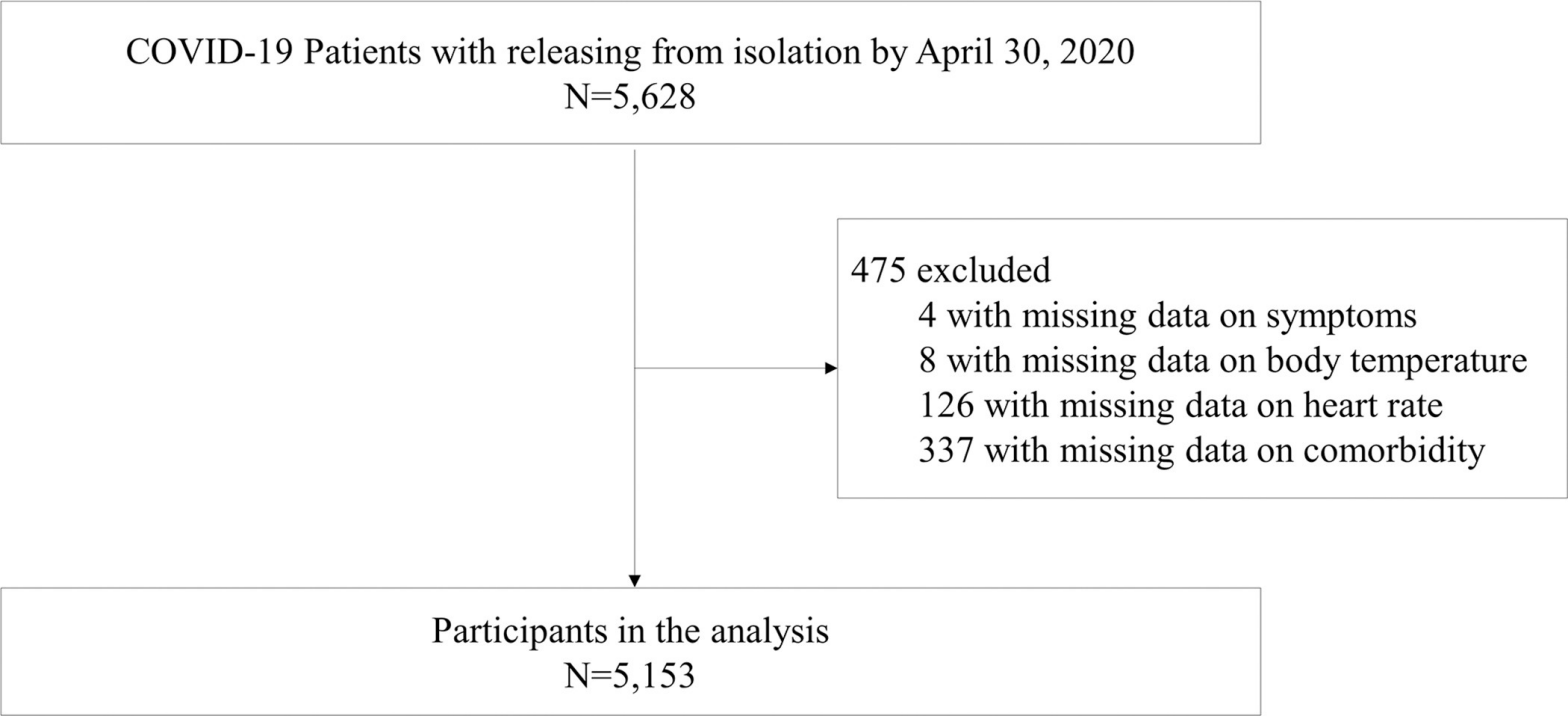
Study flow diagram.

This study was an observational, retrospective cohort study. The data were obtained from the Korea Disease Control and Prevention Agency (KDCA). The KDCA collected nationwide clinical and epidemiological data using a standardized clinical record form from hospitalized COVID-19 patients. All patients in this dataset were hospitalized because of COVID-19 diagnosis. The data are anonymized. A total of 5,628 patients confirmed to have COVID-19 and discharged from hospital up until 30, April 2020, were evaluated. Of them, 475 with missing data on main exposure variables, symptoms, and confounding variables were excluded; therefore, 5,153 subjects were analyzed (Fig 1).

### Symptoms

The principal variable was that of symptoms of COVID-19 on admission. Information on the following symptoms was provided: fever, cough, sputum, sore throat, rhinorrhea, myalgia, fatigue/malaise, dyspnea, headache, altered state of consciousness, nausea/vomiting, and diarrhea. These variables were used to define other variables to assess the association between initial symptoms and mortality. Participants asymptomatic at admission were defined as asymptomatic, and others as symptomatic. The number of symptoms on admission were also categorized as 0, 1–2, or 3+. This allowed identification of a dose-response relationship between the number of initial symptoms and mortality. In addition, we used EFA to suggest symptom combinations and estimate their factor scores, which are numerical values indicating individual relative spacings of latent factors.

### In-hospital mortality

The outcome variable was classified into discharge or death during hospitalization, and in-hospital mortality was the event.

### Covariates

KDCA provided clinical and epidemiological information at admission. Age is presented as categorical data, and for Cox regression it was divided into 60 years of age or older and 60 years of age under because there were too few deaths in the younger group. Body mass index (BMI) at admission was categorized as underweight (BMI < 18.5), normal (18.5 ≤ BMI < 25), or obesity (BMI ≥ 25). From the initial examination, the heart rate was recalculated as heart rate divided by the standard deviation. Patients’ histories of comorbidities were adjusted as binary variables; they included diabetes, hypertension, heart disease, chronic obstructive pulmonary disease (COPD), chronic kidney disease, malignancy, and dementia.

### Statistical analysis

General characteristics are presented as frequencies with percentages for categorical variables, and as means with standard deviation for continuous variables. All variables are reported by sex. An EFA was performed to identify similar grouped traits of symptoms. Because symptom variables were binary, a heterogeneous correlation matrix was computed using the *polycor* package. EFA was performed using the *psych* package. Varimax rotation was employed to clarify relationships among factors. Each symptom-related factor included several variables with a loading factor of > 0.4. A regression method was used to estimate factor scores [12]. We used four models to assess associations between initial symptoms at hospitalization and in-hospital mortality in COVID-19 patients. Model 1 included a binary variable to indicate symptomatic vs. asymptomatic. Model 2 included the number of symptoms as a categorical variable. Model 3 included indicators for individual symptoms. Model 4 included symptom combinations constructed by factor analysis. Factor scores were used as independent variables in this model to assess the effects of symptom combinations on in-hospital mortality. To identify the risk factors at admission associated with in-hospital mortality, the Cox proportional hazards model was used. Hazard ratios (HR) were estimated with 95% confidence intervals (CI). R ver. 4.0.3 software was used for statistical analysis.

### Ethics

The study protocol was approved by the Institutional Review Board of Seoul National University (No. E2009/001-008).

## Results

### General characteristics of the participants

The general characteristics of the participants are shown in Table 1. There were more female patients (58.5%) than male patients, but more male deceased (52.4%) than female deceased. The largest age group was 40–59 years (33.1%), followed by 20–39 years (29.0%). The median interval from hospital admission to release from isolation of survivors was 25 days and that of the deceased was 12 days. Most of the patients had fewer than two symptoms; 26.6% were asymptomatic, 44.8% had one to two symptoms, and 28.6% had more than three symptoms. The most prevalent symptom was cough (42.0%), followed by sputum (28.8%) and headache (16.6%). Among the deceased, the most prevalent symptom was dyspnea (47.6%), followed by cough (34.7%) and sputum (31.1%). The most common comorbidities were hypertension (22.2%) and diabetes (13.0%). Among the survivors, 2.1% were admitted to the intensive care unit (ICU) compared to 36.0% of deceased patients. Regarding disease severity, 84.2% of patients had mild disease (Class 1), and 5.6% had severe disease during hospitalization (Class 3).

**Table 1:** General characteristics of hospitalized patients with COVID-19.

	Deceased (n = 225)	Survivors (n = 4,928)	Total (N = 5,153)
**Sex (n, %)**			
Male	118 (52.4)	2,018 (40.9)	2,136 (41.5)
Female	107 (47.6)	2,910 (59.1)	3,017 (58.5)
**Age group (n, %)**			
0–19	0 (0.0)	114 (3.8)	249 (4.8)
20–39	1 (0.4)	755 (25.0)	1,493 (29.0)
40–59	16 (7.1)	1,120 (37.1)	1,705 (33.1)
60–79	95 (42.2)	813 (26.9)	1,390 (27.0)
80 +	113 (50.2)	215 (7.1)	316 (6.1)
**Median of time from hospital admission to release from isolation**	12	25	24
**Status on admission**			
Heart rate	89.6 ± 20.0	85.4 ± 14.8	85.6 ± 15.1
Body temperature (°C)	37.1 ± 0.8	36.9 ± 0.6	36.9 ± 0.6
BMI (kg/m2)			
< 18.5	16 (7.1)	227 (4.6)	243 (4.7)
18.5–25	66 (29.3)	2,591 (52.6)	2,657 (51.6)
≥ 25	44 (19.6)	1,117 (22.7)	1,161 (22.5)
Missing	99 (44.0)	993 (20.2)	1,092 (21.2)
Blood pressure			
Normal	54 (24.3)	917 (18.7)	971 (18.8)
Prehypertension	55 (24.8)	1,863 (38.0)	1,918 (37.2)
High blood pressure	113 (50.9)	2,121 (43.3)	2,234 (43.4)
**Number of symptoms**			
0	43 (19.1)	1,263 (25.6)	1,306 (25.3)
1–2	108 (48.0)	2,164 (43.9)	2,272 (44.1)
3+	74 (32.9)	1,501 (30.5)	1,575 (30.6)
**Symptoms on admission**			
Fever	67 (29.8)	765 (15.5)	832 (16.1)
Cough	78 (34.7)	2,084 (42.3)	2162 (42.0)
Sputum	70 (31.1)	1,414(28.7)	1484 (28.8)
Sore throat	11 (4.9)	785 (15.9)	796 (15.4)
Rhinorrhea	6 (2.7)	514 (10.4)	520 (10.1)
Myalgia	19 (8.4)	817 (16.6)	836 (16.2)
Fatigue/Malaise	17 (7.6)	211 (4.3)	228 (4.4)
Dyspnea	107 (47.6)	514 (10.4)	621 (12.1)
Headache	12 (5.3)	844 (17.1)	856 (16.6)
Altered state of consciousness	20 (8.9)	11 (0.2)	31 (0.6)
Nausea/vomiting	15 (6.7)	224 (4.5)	239 (4.6)
Diarrhea	17 (7.6)	431 (8.7)	448 (8.7)
**Comorbidities**			
Diabetes mellitus	93 (41.3)	575 (11.7)	668 (13.0)
Hypertension	138 (61.3)	1,006 (20.4)	1144 (22.2)
Chronic heart disease	37 (16.4)	178 (3.6)	215 (4.2)
Asthma	13 (5.8)	109 (2.2)	122 (2.4)
COPD	8 (3.6)	31 (0.6)	39 (0.8)
Chronic kidney disease	16 (7.1)	38 (0.8)	54 (1.0)
Malignancy	20 (8.9)	122 (2.5)	142 (2.8)
Chronic liver disease	6 (2.7)	74 (1.5)	80 (1.6)
Rheumatic disease/Autoimmune disease	3 (1.3)	34 (0.7)	37 (0.7)
Dementia	75 (33.3)	144 (2.9)	219 (4.2)
**ICU use status during hospitalization**			
ICU care	81 (36.0)	104 (2.1)	185 (3.6)
Non ICU care	144 (64.0)	4,796 (97.9)	4940 (95.5)
**Maximum severity during hospitalization** *			
Class 1	-	4,338 (88.5)	4,338 (84.2)
Class 2	-	501 (10.2)	501 (9.7)
Class 3	-	62 (1.3)	287 (5.6)

Abbreviations: BMI = body mass index; COPD = chronic obstructive pulmonary disease; ICU = intensive care unit.

* Class 1, no limitation of daily activities or limitation of daily activities but no need for supplemental oxygen therapy; Class 2, need for supplemental oxygen therapy via nasal cannula or facial mask; Class 3, need for high-flow supplemental oxygen therapy or non-invasive mechanical ventilation or invasive mechanical ventilation or multi-organ failure or need for extracorporeal membrane oxygenation (ECMO) therapy or death.

### Factor analysis of symptoms

Three factors were identified by EFA, and their cumulative variance was 35.7% (S1 Table). Factor 1 encompassed cough, sputum, and rhinorrhea (cold-like symptoms) and accounted for 13.2% of the total variance. Factor 2 included neurological symptoms such as myalgia, fatigue/malaise and headache, and gastrointestinal symptoms such as nausea/vomiting and diarrhea (12.0% of the total variance). Factor 3 consisted of dyspnea and altered state of consciousness and explained 10.5% of the total variance. The symptoms related to factor 3 were moderate/severe. A correlation matrix of the symptoms is shown in S2 Table.

### Associations between initial symptoms and in-hospital mortality

**Fig 2:**
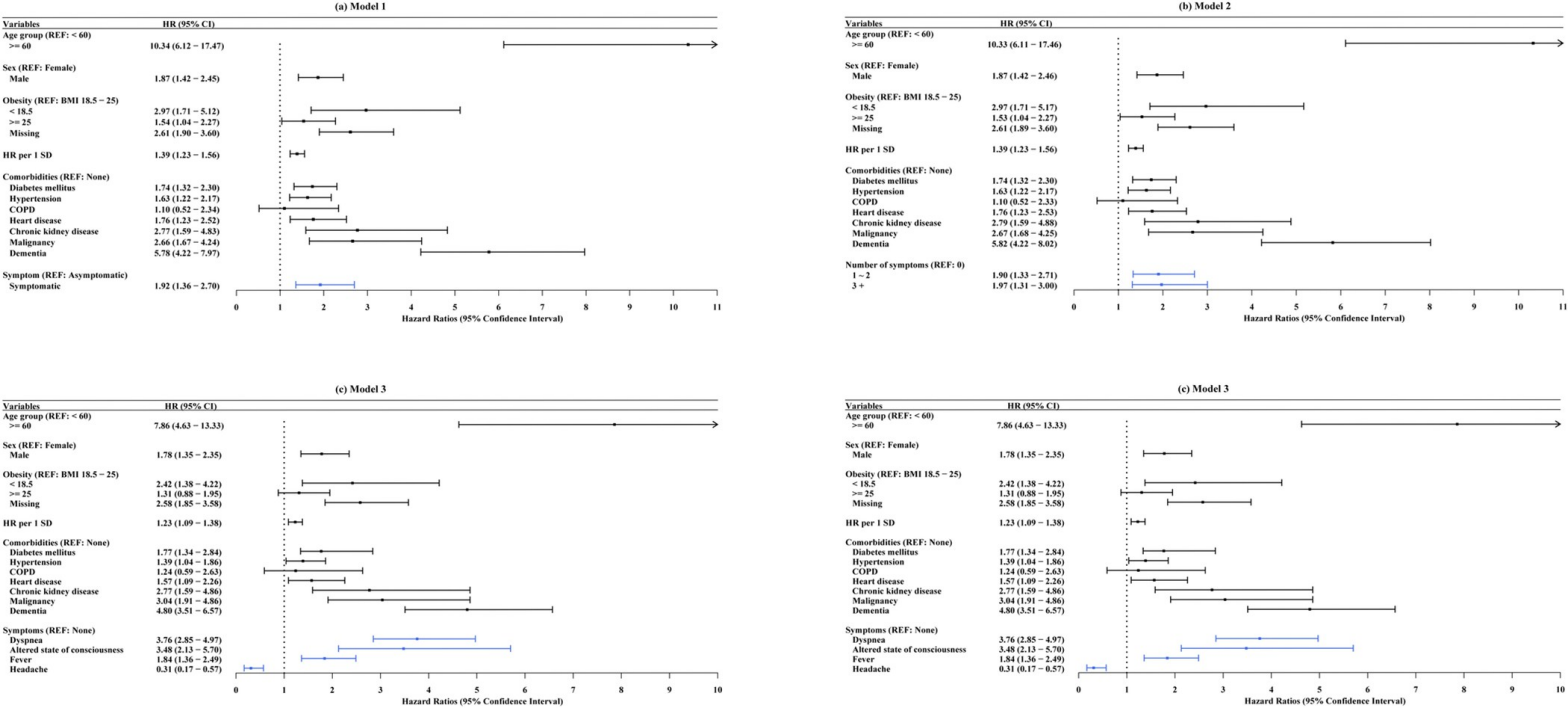
Multivariate cox regression of hazard ratios of in-hospital mortality in COVID-19 patients. (a) Model 1: HR of symptom presence adjusting for age group, sex, obesity, heart rate, and comorbidities; (b) Model 2: HR of the number of symptoms adjusting for age group, sex, obesity, heart rate, and comorbidities; (c) Model 3: HR of each significant symptom adjusting for age group, sex, obesity, heart rate, and comorbidities; (d) Model 4: HR of each factor score adjusting for age group, sex, obesity, heart rate, and comorbidities. Abbreviations: REF = reference; HR = heart rate; SD = standard deviation; BMI = body mass index; COPD = chronic obstructive pulmonary disease.

Our findings were shown in Fig 2. Age over 60 years, male, underweight, obese, and comorbidities were significantly associated with in-hospital mortality in all models. Regarding all models, male sex was a significantly stronger risk factor than female sex. Taking normal as the reference, the underweight and obese groups had significantly higher HRs for death in all models except Model 3. The higher the HR at admission, the higher the risk for death. Patients with diabetes, hypertension, heart disease, chronic kidney disease, malignancy, and dementia had higher HRs than those without such diseases, whereas the HR of COPD was not significant in all models. In model 1, symptomatic patients had a higher HR for mortality than asymptomatic patients (HR 1.92, CI 1.36–2.70) after adjusting for covariates. In model 2, the higher the number of symptoms, the higher the risk of mortality. Patients with one or two symptoms (HR 1.90, CI 1.33–2.71) or three or more symptoms (HR: 1.97, CI: 1.31–3.00) had a higher risk for mortality than asymptomatic patients. In model 3, patients with dyspnea (HR: 3.76, CI: 2.85–4.97) had the highest mortality risk. Also, altered state of consciousness (HR 3.48, CI 2.13–5.70) and fever (HR:1.84, CI: 1.36–2.49) were associated with death. Headache, however, was associated with a lower risk for death (HR 0.31, CI 0.17–0.57). Model 4 considered the grouped traits of the symptoms using the factor scores as independent variables. Patients with higher factor scores have more factor-related symptoms, and higher factor loadings indicate higher factor scores. Patients with higher factor 1 scores have a higher risk for death (HR: 1.14, CI:1.01–1.30). Factor 2 symptoms (neurological and gastrointestinal symptoms) were associated with a lower risk for death (HR: 0.83, CI: 0.71–0.96). Patients with a higher factor score for factor 3 were at an elevated risk for death (HR 1.25, CI 1.19–1.31).

**Fig 3:**
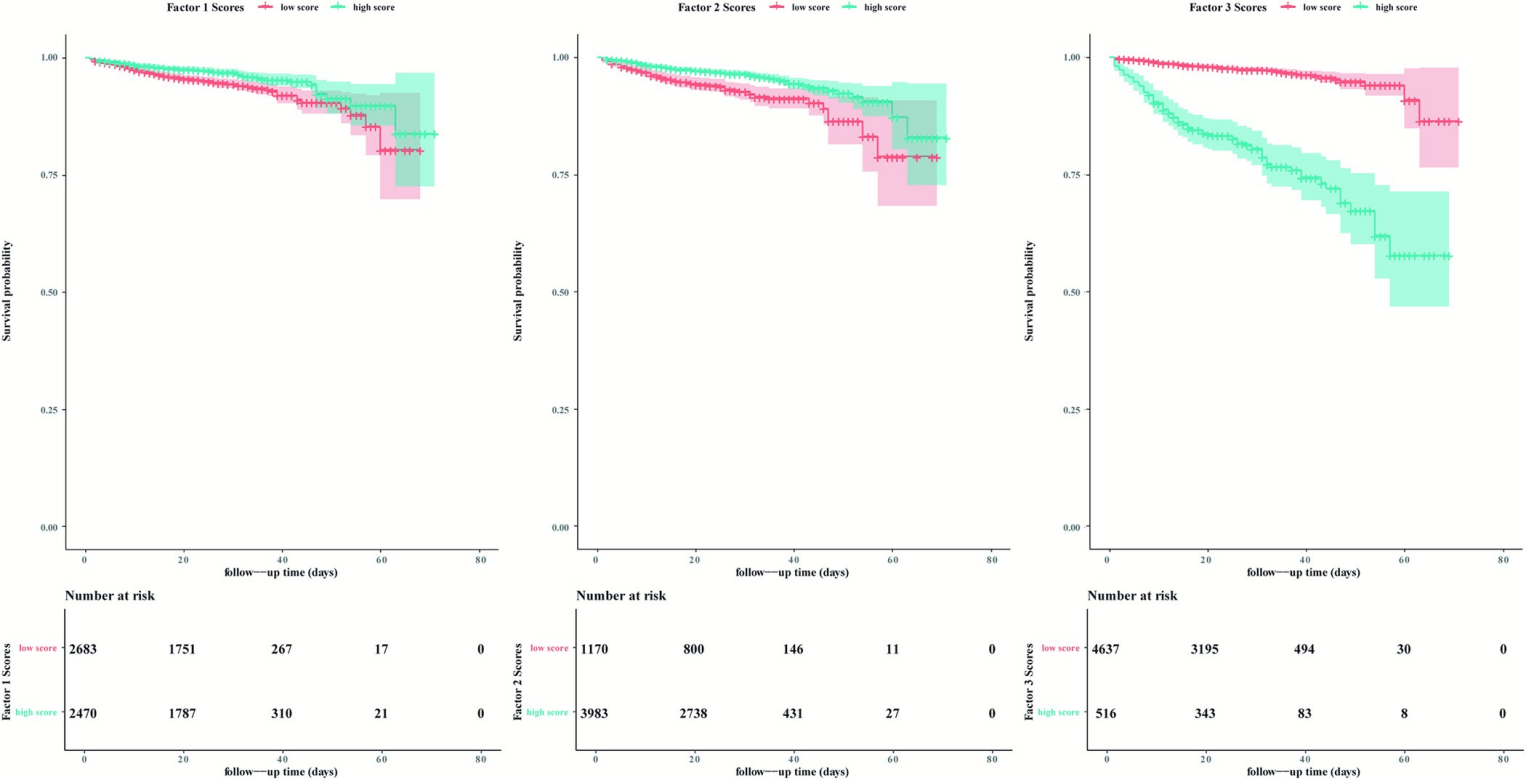
Kaplan-Meier plot of symptom-related factor scores of in-hospital mortality in COVID-19 patients. Survival probabilities of (a) factor 1 scores; (b) Factor 2 scores; (c) Factor 3 scores.

The Kaplan-Meier plots for COVID-19 mortality are shown in Fig 3. To generate the Kaplan-Meier plot, three factor score variables were dichotomized at the optimal cutoff point using the ‘maxstat’ package in R [13]. For factors 1 and 2, the higher-score group had a lower survival probability, and for factor 3, the lower-score group had a higher survival probability.

Table 2 lists the unadjusted and adjusted models of Cox regression. As shown in Fig 3, in the unadjusted model, the hazard ratios of factors 1 and 2 were lower than 1 and that of factor 1 was nonsignificant, whereas the hazard ratio of factor 3 was significantly higher than 1. After adjusting for covariates, the hazard ratio of factor 1 was higher than 1 and significant, whereas the values of the other factors tended to be identical to those prior to adjustment.

**Table 2:** Unadjusted and adjusted models of Cox regression.

	Unadjusted Model	Adjusted Model 1^1)^	Adjusted Model 2^2)^
Factor 1 score	0.99 (0.87–1.12)	1.00 (0.88–1.13)	1.14 (1.01–1.30)
Factor 2 score	0.72 (0.62–0.83)	0.75 (0.65–0.87)	0.83 (0.71–0.96)
Factor 3 score	1.42 (1.36–1.48)	1.29 (1.23–1.35)	1.25(1.19–1.31)

^**1)**^Adjusted model 1 was adjusted for age, sex, obesity, and heart rate

^**2)**^adjusted model 2 was adjusted for the variables in model 1, plus comorbidities such as diabetes mellitus, hypertension, COPD, heart disease, chronic kidney disease, malignancy and dementia.

## Discussion

We evaluated characteristics of COVID-19 patients to estimate associations between symptoms at admission and in-hospital mortality. Cough was the most frequent symptom at admission. Among symptomatic patients, the highest mortality rate was 64.5% among those with altered state of consciousness.

Symptomatic patients had a higher HR for death than asymptomatic patients, and the HR for three or more symptoms was higher than that for one or two symptoms, with asymptomatic patients as the reference group. Fever, dyspnea, and altered state of consciousness increased the risk for death whereas headache decreased the risk. Three symptom combinations were suggested: factor 1 constituted cold-like symptoms (cough, sputum, and rhinorrhea), factor 2 constituted neurological and gastrointestinal symptoms (myalgia, fatigue/malaise, headache, nausea/vomiting and diarrhea), and the more severe symptoms of COVID-19 were dyspnea and altered state of consciousness, which grouped as factor 3. Factors 1 and 3 increased the risk for death, whereas factor 2 decreased the risk.

Dyspnea is consistently reported as a risk factor for mortality in COVID-19 patients [14–18]. The impact of fever on death is controversial. Fever was reported to be a significant risk factor for in-hospital mortality in two studies [19,20], but to decrease the risk for mortality among COVID-19 patients in another [15]. Yet other studies have reported that fever is not associated with mortality [14,16–18]. Although headache is reportedly not significantly associated with mortality [15], it has been suggested to decrease the risk in others [16,18,21], likely because headache is typically accompanied by other mild symptoms [21]. We found that headache decreased the risk for mortality. Also, altered state of consciousness was a risk factor for mortality in COVID-19 patients. However, the symptoms are not independent in this regard, because of other underlying pathogenic mechanisms. Therefore, evaluation of the effects of symptom combinations on mortality are required.

The natural history of COVID-19 consists of three phases [22]. The first phase is accompanied by mild symptoms, such as cold-like symptoms, which progress to moderate symptoms. The second, or pulmonary, phase is mediated by unknown factors and induces pneumonia-like symptoms. Severe hypoxemia is a symptom of COVID-19 pneumonia. During this phase, the prognosis is dependent on disease severity. Patients who deteriorate and progress to the third phase typically experience hyperinflammation and lung sepsis. Such persons require ICU care and few recover [22]. Generally, patients with mild symptom combinations may show signs of recovery after 1 week, but some may have persistent symptoms or rapidly worsen [23].

The symptoms related to factors 1 and 2 are prodromal and most are mild [24]. Factor 1 symptoms (cough, sputum, and runny nose) are typically the first symptoms of COVID-19. In this study, patients with cough or sputum had higher factor scores for factor 1 and had a higher risk for mortality. If the disease progresses, sputum production may increase and changes in its composition indicate disease exacerbation [25]. Cough can also worsen and progress to pneumonia. Patients with high factor 2 scores had neurological and gastrointestinal symptoms rather than fever or respiratory symptoms. These symptoms tended to be present together. Of these symptoms, headache made the greatest contribution to the highest factor 2 score. Headache is reportedly linked to a more effective immune response and could be associated with lower mortality [21,26]. Gastrointestinal symptoms were associated with a lower risk for mortality or severity [27–29], but the association was not significant in a meta-analysis [30]. Immunoglobulin A, which neutralizes SARS-CoV-2, reduces the risk for mortality in patients with gastrointestinal symptoms [29]. Inflammatory reactions in the lungs to SARS-CoV-2 infection cause pulmonary edema, reduced gas exchange, and dyspnea. If the immune response is not controlled in patients with aggravated COVID-19, the levels of cytokines such as IL-6 increase, possibly inducing a cytokine storm and causing serious lung damage and death [14]. This explains why the risk for mortality in COVID-19 patients increased with increasing factor 3 score.

It has been suggested that symptoms corresponding to factor 1 occur first, followed by those of factor 2 [31]. We hypothesize that patients with factor 1 symptoms could progress to factor 2 symptoms and, thereafter, either recover or progress to factor 3 symptoms. Patients with factor 1 symptoms could deteriorate and die upon developing factor 3 symptoms, whereas factor 1 patients could recover their immune function and show factor 2 symptoms. Factor 1 and 2 symptoms occur early in COVID-19 patients, but the prognosis differed according to symptom combination. However, because symptoms were investigated once at admission, further study of the effect of changes in symptoms and their severities over time on the risk for mortality is warranted.

Male sex, older age, underweight, obesity, and higher HR were predictive of mortality among COVID-19 patients. This is consistent with reports that hypertension, diabetes, heart disease [32–34], chronic kidney disease [35], malignancy [36,37], and dementia [38] are the most common comorbidities of COVID-19 and are associated with a higher risk for mortality. COPD was not significantly associated with the risk for mortality, as reported previously [39].

Most individual symptoms were not associated with in-hospital mortality, as in previous studies. However, the associations between symptom combinations and mortality in COVID-19 patients were significant, and the effect on in-hospital mortality differed according to symptom combination. Therefore, assessing symptoms individually and in combination will facilitate early and rapid identification of patients at risk of death, enabling immediate intervention and increasing the survival rate. Further research on symptom combinations, taking into account the duration of symptoms and changes over time as predictors of death, is needed.

The strength of this study is that it is the first to identify associations between symptom combinations and in-hospital mortality. In addition, the data were the Korean nationwide data of COVID-19 patients. Because most people confirmed with COVID-19 during the early pandemic in South Korea were hospitalized, our results can be generalized. The study had several limitations. We examined symptoms at admission, and the timing of admission may vary by patient. However, COVID-19 diagnosis was made within 5 days of symptom onset in most cases in South Korea during the study period [40], because of a preemptive testing strategy. Therefore, symptoms at admission were mostly those of the early phase of COVID-19. Secondly, because the data were from the early stage of the pandemic, prior to the emergence of dangerous SARS-CoV-2 variants, a database of recently confirmed COVID-19 cases is needed to assess associations between symptom combinations and death. In addition, individuals excluded from the analysis due to missing values made up a higher proportion of asymptomatic patients but had a similar mortality rate. Therefore, exclusion of those with missing data would not have biased the results. Lastly, Because we evaluated statistical associations, the results do not imply causation [41].

Vaccination for COVID-19 has been introduced, and resurgence of COVID-19 due to novel variants has occurred repeatedly. COVID-19 disease severity and mortality have been reduced by vaccines and increased by novel variants. However, no study has evaluated the ability of symptom combinations to compare the changes. Because symptoms are not independent, identifying latent factors that represent underlying pathophysiologic processes may shed light on the effects of vaccination and breakthrough infection and the characteristics of infection with novel variants. Whether these symptom combinations affect the post-COVID condition warrants further investigation.

## Conclusion

In conclusion, COVID-19 symptom combinations were related to in-hospital mortality rate. The combination of dyspnea and altered state of consciousness was associated with the highest risk for mortality, followed by cold-like symptoms. Neurological/gastrointestinal symptoms were negatively associated with mortality. Our findings show that symptom combinations can be used to triage patients and to guide allocation of limited medical resources. Creation of symptom combinations by factor analysis methods could be applied to explore the clinical characteristics of COVID-19 after the emergence of novel variants or introduction of vaccines, and to investigate associations between symptom combination and post-COVID conditions.

## Supporting information

S1 Table Rotated factor loadings for symptom variables. (DOCX)

S2 Table Polychoric correlation matrix of symptom variables. (DOCX)
