# Supplementary material for: Associations of symptom combinations with in-hospital mortality of coronavirus disease-2019 patients using South Korean National data
Source: PLoS One. 2022 Aug 26;17(8):e0273654. doi: 10.1371/journal.pone.0273654 (PMC9417015; doi:10.1371/journal.pone.0273654)
Supplement: S1 Table — (DOCX) [file pone.0273654.s001.docx]

**S1 Table. Rotated factor loadings for symptom variables.**

|  | **Factor 1** | **Factor 2** | **Factor 3** |
| --- | --- | --- | --- |
| Fever |  | 0.266 | 0.103 |
| Cough | **0.841** | 0.157 |  |
| Sputum | **0.741** | 0.137 | 0.155 |
| Sore throat | 0.255 | 0.233 | -0.201 |
| Rhinorrhea | 0.304 | 0.203 |  |
| Myalgia | 0.161 | **0.518** |  |
| Fatigue/Malaise | 0.146 | 0.312 | 0.249 |
| Dyspnea | 0.227 | 0.173 | **0.574** |
| Headache | 0.166 | **0.632** |  |
| Altered state of consciousness | -0.139 |  | **0.807** |
| Nausea/vomiting |  | **0.445** | 0.311 |
| Diarrhea |  | **0.482** | 0.185 |
|  |  |  |  |
| **Sum of squared loadings** | 1.582 | 1.440 | 1.259 |
| **Proportion variance** | 0.132 | 0.120 | 0.105 |
| **Cumulative variance** | 0.132 | 0.252 | 0.357 |
