# Supplementary material for: Associations of symptom combinations with in-hospital mortality of coronavirus disease-2019 patients using South Korean National data
Source: PLoS One. 2022 Aug 26;17(8):e0273654. doi: 10.1371/journal.pone.0273654 (PMC9417015; doi:10.1371/journal.pone.0273654)
Supplement: S2 Table — (DOCX) [file pone.0273654.s002.docx]

**S2 Table. Polychoric correlation matrix of symptom variables**

|  | Fever | Cough | Sputum | Sore throat | Rhinorr-hea | Myalgia | Fatigue/Malaise | Dyspnea | Headache | Altered state of concious-ness | Nausea/vomiting | Diarrhea |
| --- | --- | --- | --- | --- | --- | --- | --- | --- | --- | --- | --- | --- |
| Fever | 1.0000 | 0.1556 | 0.0738 | 0.0016 | -0.0319 | 0.2159 | 0.2165 | 0.2101 | 0.1619 | 0.0439 | 0.1613 | 0.1016 |
| Cough | 0.1556 | 1.0000 | 0.6549 | 0.2285 | 0.2756 | 0.2083 | 0.1735 | 0.2706 | 0.2273 | -0.0595 | 0.1492 | 0.1815 |
| Sputum | 0.0738 | 0.6549 | 1.0000 | 0.1835 | 0.2716 | 0.1959 | 0.2188 | 0.2712 | 0.2019 | 0.0350 | 0.1441 | 0.1661 |
| Sore throat | 0.0016 | 0.2285 | 0.1835 | 1.0000 | 0.2452 | 0.2527 | 0.0721 | -0.0120 | 0.1745 | -0.1696 | 0.0007 | 0.0866 |
| Rhinorrhea | -0.0319 | 0.2756 | 0.2716 | 0.2452 | 1.0000 | 0.1573 | 0.1419 | 0.0233 | 0.1986 | -0.0084 | 0.0208 | 0.1517 |
| Myalgia | 0.2159 | 0.2083 | 0.1959 | 0.2527 | 0.1573 | 1.0000 | 0.2211 | 0.1119 | 0.3624 | 0.0428 | 0.1468 | 0.2603 |
| Fatigue/Malaise | 0.2165 | 0.1735 | 0.2188 | 0.0721 | 0.1419 | 0.2211 | 1.0000 | 0.1794 | 0.1559 | 0.2100 | 0.2625 | 0.1751 |
| Dyspnea | 0.2101 | 0.2706 | 0.2712 | -0.0120 | 0.0233 | 0.1119 | 0.1794 | 1.0000 | 0.1529 | 0.4528 | 0.2675 | 0.1573 |
| Headache | 0.1619 | 0.2273 | 0.2019 | 0.1745 | 0.1986 | 0.3624 | 0.1559 | 0.1529 | 1.0000 | -0.0564 | 0.2986 | 0.3011 |
| Altered state of conciousness | 0.0439 | -0.0595 | 0.0350 | -0.1696 | -0.0084 | 0.0428 | 0.2100 | 0.4528 | -0.0564 | 1.0000 | 0.2546 | 0.1792 |
| Nausea/vomiting | 0.1613 | 0.1492 | 0.1441 | 0.0007 | 0.0208 | 0.1468 | 0.2625 | 0.2675 | 0.2986 | 0.2546 | 1.0000 | 0.3527 |
| Diarrhea | 0.1016 | 0.1815 | 0.1661 | 0.0866 | 0.1517 | 0.2603 | 0.1751 | 0.1573 | 0.3011 | 0.1792 | 0.3527 | 1.0000 |
